# An ingredient co-occurrence network gives insight into e-liquid flavor complexity

**DOI:** 10.18332/tid/175955

**Published:** 2024-01-10

**Authors:** Jeroen L. A. Pennings, Ina M. Hellmich, Sanne Boesveldt, Reinskje Talhout

**Affiliations:** 1 Centre for Health Protection, National Institute for Public Health and the Environment (RIVM), Bilthoven, The Netherlands; 2 Division of Human Nutrition and Health, Wageningen University, Wageningen, The Netherlands

**Keywords:** e-liquids, flavor ingredients, EU-CEG, attractiveness, dimethyl sulfide

## Abstract

**INTRODUCTION:**

Part of the appeal of e-cigarettes lies in their available flavors. To achieve attractive flavors, e-liquids contain many different flavoring agents, which allow many flavoring combinations. To advance our knowledge of e-liquid flavors and compositions and to evaluate the effect of legislation, we determined whether there are ingredient combinations that are frequently used together.

**METHODS:**

We used e-cigarette ingredient data from the European Common Entry Gate system (EU-CEG) as available on 31 December 2022.

**RESULTS:**

In e-liquids, we found 214 ingredient pairs with a co-occurrence odds ratio greater than 10. Together, these consisted of 62 unique ingredients. Network analysis revealed that ingredients were grouped together based on their flavor and/or chemical structure. We identified two densely connected regions (clusters) in the network. One consisted of six ingredients with sweet-vanilla-creamy flavors. The second cluster consisted of 13 ingredients. While some of these have fruity flavors, others, such as alkyl carboxylic acids and dimethyl sulfide, are known to have unpleasant flavors. Additional data and literature analyses indicated that alkyl carboxylic acids can contribute to a creamy and sweet-fruity taste, whereas dimethyl sulfide can contribute to a more refined fruity taste.

**CONCLUSIONS:**

These results exemplify that the flavor of e-liquids is not just the sum of its parts. Big data analyses on product data can be used to detect such patterns, but expert knowledge and additional data are needed for further interpretation. Monitoring of e-liquid flavors as well as ingredients will remain important to regulate e-liquid product attractiveness.

## INTRODUCTION

E-cigarettes are used by smokers and non-smokers. Many adult e-cigarette users are smokers who use e-cigarettes as a tool for smoking cessation or reduction, and there is some evidence for the effectiveness of e-cigarettes in that^1^. However, e-cigarettes are still harmful and addictive, and, additionally, they expand the nicotine market^2^. For example, e-cigarettes are also becoming increasingly popular among young people, including adolescents who do not (yet) use tobacco products^3-5^.

An important reason why both young people and adults are attracted to e-cigarettes is that these are available in a wide range of appealing flavors^3,6,7^. Recently, the Dutch government announced a ban on all e-liquid flavors other than tobacco, to protect the health of young people while keeping e-cigarettes available for smokers who want to quit^8^. This flavor ban took effect on 1 January 2023, albeit with a one-year transition period during which current e-liquids are still allowed on the market^9^. The flavor ban will be implemented by enforcing a restrictive set of 16 allowed flavor ingredients whose flavor is associated with tobacco or tobacco smoke, and that do not pose a known health hazard^9,10^. Various kinds of bans on flavors and/or flavoring agents are also in place in several other countries. At the time this article was written, these comprised Canada, China, Denmark, Estonia, Finland, Lithuania, Hungary, New Zealand, the Philippines, Ukraine, and the United States.

Usually, flavorings do not come in isolation, and the combinations in which flavorings are presented can largely determine how their flavor is perceived. For example, a pinch of salt mixed into the batter of a cake can enhance the sweetness of sugar^11^. On average, 10 ± 15 flavoring ingredients are used in e-liquids reported for the Dutch market, with the number and type of flavorings used depending on their flavor category (such as tobacco, menthol, or fruit)^12^.

As multiple flavorings are mixed together in e-liquids, flavor interactions can occur, leading to the perception of the mixture being different from (the sum of) the perception of its individual ingredients. These interactions can have varying effects on flavor intensity, flavor quality, and flavor appeal. Some flavors blend, with the result that the unified perception of the mixture is qualitatively different from each of its components. For example, Moio et al.^13^ sniffed all individual components of a cheese aroma separated by gas chromatography and found that none of those had a cheesy aroma when presented in isolation^13^. Burghard and Kuznicki^14^ found that ‘coffee aroma is contributed to by several hundreds of compounds, a great many of which do not smell anything like coffee’^14^. For e-liquids, this could mean that a flavor different from tobacco could potentially be mixed through flavor interaction between the permitted compounds. This would defy the purpose of a flavor ban.

Another interesting characteristic of flavors in mixtures is that an individual ingredient, which might be perceived as unpleasant on its own, can actually boost the overall appeal or pleasantness of the mixture. For example, the floral scent of jasmine contains considerable amounts of indole, a chemical compound that by itself is rated as unpleasant but enhances the hedonic potency of the mixture^15^. Other interactions that can occur relate to the suppression of unpleasant flavors, for example, sweet compounds, are often added to decrease the perceived intensity of bitter compounds^16^. In e-liquids, flavoring ingredients might, for example, be added to suppress the unpleasant taste of nicotine. Finally, flavor interactions can have pharmacological and/or toxic consequences. For example, menthol, as well as non-menthol cooling agents, generate cooling sensations that mask nicotine’s harshness, which has implications for inhalation and nicotine uptake^17,18^.

Considering the examples given above, additional insights into the sensory effects of flavorings and flavoring combinations will be useful to better interpret e-liquid compositions and how these translate into a flavor. Furthermore, such insights can be used to better evaluate the effect of regulations such as flavor bans in several countries. In order to advance our understanding of e-liquid flavor and product composition, we examined if we could identify ingredients that are often used in combination and whether such ingredient combinations could give us insight into e-liquid composition and flavor.

## METHODS

E-cigarette ingredient data were obtained from the European Common Entry Gate system (EU-CEG)^19^. This is a database in which manufacturers and importers are legally obliged to provide information about the composition and other properties of the tobacco products and e-cigarettes they market in each European Union Member State^20^. We used data as available for active products in the Dutch section of the EU-CEG system at the end of 31 December 2022 (before the flavor ban officially took effect). EU-CEG distinguishes several types of e-cigarette products, of which we only used data for product types containing e-liquid, i.e. e-liquid refills and disposable, rechargeable, and refillable e-cigarettes that contain e-liquid. Other product types, such as device-only e-cigarettes or product parts, were excluded from further analysis. Product data were exported as tab-delimited text, and further statistical analyses were carried out in R (version 4.2.0) and Microsoft Excel.

Using Excel, the list of ingredients was curated, and some ingredients were merged to remove redundancy. This was done for cases where ingredients were essentially the same, including their flavor properties, but one form was notified as: 1) a stereoisomer (e.g. menthol and L-menthol); 2) a tautomer (e.g. the 3-methyl-1,2-cyclopentanedione and 2-hydroxy-3-methylcyclopent-2-en-1-one forms of cyclotene); 3) a hydrate form (e.g. citric acid and citric acid monohydrate); or 4) a hemiacetal form (e.g. vanillin and vanillin propylene glycol acetal, the latter of which gets formed by vanillin in the e-liquid matrix). Data for ingredients that were not used in at least 1% of at least one of the four product types studied, were discarded because their use was considered too rare to be informative. Also, data for propylene glycol, glycerol, and water were excluded because their presence is considered so common that it is trivial. Besides flavorings, the resulting data set also contained ingredients with (presumably) other functions, such as nicotine, sorbic acid (preservative), or trisodium citrate (pH modifier).

Ingredient co-occurrence analyses were performed in R. For all possible ingredient pairs, we made a cross-tabulation matrix to determine the percentage of products in which they co-occurred as well as the odds ratio using the Fisher’s test of the R function. An example of such a calculation is given in Supplementary file Example 1, and a more in-depth statistical explanation is given in Szumilas^21^. If ingredients co-occurred in at least 3% of the products and at least 10 products (whichever of these values is larger) with an odds ratio of at least 10, their cooccurrence was considered significant.

To determine the robustness of our significance criteria, i.e. to determine the degree of chance findings, the cross-tabulation analysis was repeated for data where the ingredient presence was randomly redistributed among the e-cigarette products. The analyses in this paragraph were performed for each product type separately. The lists of ingredient pairs for the different product types were compared using Venn diagrams using R software and the *limma* package.

Ingredient pairs with significant co-occurrence were saved in a file that was imported in Cytoscape 22 (version 3.9.1) for network analysis and visualization. Cytoscape is a software platform for analyzing biomolecular networks using tools such as two-dimensional layout visualization and annotating the function of network components by shapes and/or colors. Each ingredient pair was visualized as a connection (network edge) between two ingredients (network nodes). The network layout was visualized using the ‘organic’ layout algorithm. This algorithm treats connected ingredients as if they were connected by a kind of metal springs so that connected ingredient pairs attract each other if they are farther apart or repel each other if they are closer together. The resulting forces lead to all ingredients being arranged in a two-dimensional graph in a way that minimizes the overall force^22,23^. This was followed by minor manual adjustments to the layout to improve legibility. To identify densely connected regions (clusters) within a network, we used the MCODE^24^ app with default settings.

Ingredient flavor descriptions were obtained from the Leffingwell database^25^ which contains flavor data relevant to the food, beverage, and tobacco industry.

## RESULTS

### Co-occurrence statistics

For each of the four e-cigarette product types, the number of products, the number of significant ingredient co-occurrence pairs, and the number of ingredients that comprise these combinations are listed in Table 1. The number of products was highest for e-liquids (n=33179), and here the largest number of significant ingredient pairs was found (n=214). Fewer significant ingredient pairs were found for disposable (n=13) and rechargeable (n=43) e-cigarettes, and none was found for refillable e-cigarettes. For all product types, robustness testing with randomized data did not result in any significant ingredient pairs.

**Table 1:** Co-occurrence analysis summary of e-cigarette ingredient data from the European Common Entry Gate system (EU-CEG) as available on 31 December 2022

*Product type*	*E-liquid*	*Disposable*	*Rechargeable*	*Refillable*
**Co-occurrence statistics**				
Number of products	33179	10970	248	51
Number of significant ingredient pairs (network edges)	214	13	43	0
Number of unique ingredients (network nodes)	62	19	41	0
**Network parameters**				
Average number of connections per node	7.5	1.8	2.5	NA
Average distance between two nodes (characteristic path length)	2.6	2.4	3.6	NA
Largest distance between two nodes (network diameter)	7	5	8	NA

**Figure 1:**
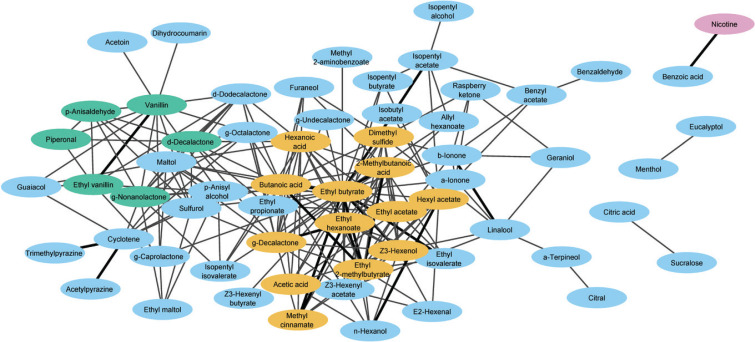
E-liquid ingredient network. Ingredient pairs that have significant co-occurrence are shown as network connections. The network was visualized using Cytoscape. Green, MCODE cluster A; orange, MCODE cluster B; blue, other flavorings; pink, nicotine. Bold lines indicate ingredient pairs with significant cooccurrence in multiple product types

With Cytoscape software, ingredient pairs for each product type were visualized as a network. This resulted in ingredient networks for e-liquids (Figure 1), disposables (Supplementary file Figure 1), and rechargeable e-cigarettes (Supplementary file Figure 2), respectively.

The e-liquid ingredient network consisted of 62 ingredients with 214 connections among them (Figure 1 and Table 1). For most of the ingredients in the network, their function is notified in EU-CEG as flavoring (EU-CEG dictionary term: flavor and/or taste enhancer), with nicotine as the only exception. Leffingwell database flavor descriptions indicated that most of the flavorings have a fruity or sweet flavor and odor, with some exceptions, such as menthol. Overall, ingredients that end up near each other in the network often have similar flavoring and/or chemical properties, for example, esters, lactones, terpenes, or pyrazines.

The network consisted of three isolated pairs and a larger network. This larger network had a diameter of 7; in other words, any two ingredients in the network are linked by a path of ≤7 steps. The network was internally well-connected; the average number of connections per ingredient was 7.5, ranging from one for 15 ingredients to 30 for ethyl butyrate. The number of connections was weakly correlated to its prevalence of use (Pearson’s R=0.27). However, additional analyses showed that some ingredients with high use prevalence (e.g. benzyl alcohol, which was used in 14.1 % of the e-liquids, or trimethyl isopropyl butanamide (WS-23), used in 10.3%) did not co-occur with any other ingredients, which indicates that this overall trend also has some clear exceptions.

Because some areas in the e-liquid ingredient network appeared to be more densely connected, we determined if we could identify clusters, which in this context means network regions for which the ingredients are more densely connected among themselves compared to the overall network. This resulted in two clusters (Table 2 and Supplementary file Figure 3). The first of these clusters (cluster A) consisted of 12 connections and 6 ingredients whose flavor descriptions typically contain words such as ‘sweet’, ‘vanilla’, and ‘creamy’. Of these ingredients, vanillin and ethyl vanillin had the highest use prevalence as well as median concentration (Table 2). Cluster B consisted of 56 connections and 13 ingredients. This cluster was more diverse, both from a chemical as well as a sensory point of view. Six of the ingredients are esters with mainly sweet and fruity flavors. Four ingredients are alkyl carboxylic acids with a sour and/or cheesy flavor, which by themselves can be considered unattractive flavors for e-liquids. Two of the remaining ingredients (Z-3-hexanol and gamma-decalactone) have a pleasant green and a coconut-peach flavor, respectively. The final ingredient in cluster B, dimethyl sulfide, had the lowest use prevalence as well as the lowest median concentration of the flavorings in cluster B. It has a ‘pungent, cooked vegetable-like’ unpleasant flavor in isolation but also acts as a component in fruit and rose flavors, according to the Leffingwell database (Table 2). Although the size and composition of both clusters A and B changed somewhat upon using non-default settings in the MCODE algorithm, the main finding that cluster B consisted of ingredients with both pleasant and unpleasant flavors remained valid (data not shown).

**Table 2:** Ingredients in the network MCODE clusters, based on the assessment of e-cigarette ingredient data from the European Common Entry Gate system (EU-CEG) as available on 31 December 2022

*Ingredient name*	*Flavor description (Leffingwell)*	*Smoke taste and aroma (Leffingwell)*	*E-liquid use prevalence %*	*Median concentration (mg/mL)*
**Cluster A**				
Vanillin	Powerful, creamy, vanilla-like odor and sweet taste	Sweet, vanilla	34.8	0.95
Ethyl vanillin	Intense, sweet, vanilla-like odor; creamy vanilla taste	Sweet, vanilla	18.2	1.15
Piperonal	Sweet, floral-cherry (heliotrope); sweet cherry-vanilla taste	Sweet, floral, cherry, vanilla	8.1	0.14
*p*-Anisaldehyde	Floral, hay-like odor; sweet anisic-vanilla-fruity herbaceous	Sweet, floral, hay, anise	8.0	0.08
delta-Decalactone	Sweet, creamy, milky, peach, nut; peach, buttery in dilution	Sweet, smooth, buttery	9.3	0.04
gamma-Nonalactone	Strong, fatty, coconut odor and taste	Coconut, fatty	7.0	0.10
**Cluster B**				
Ethyl butyrate	Ethereal, fruity odor; buttery-pineapple-banana, ripe fruit and juicy notes	Sweet, fruity, winey	35.7	0.79
Ethyl acetate	Ethereal, sharp, wine-brandy-like odor	Weak fruity, chemical	25.5	0.31
Ethyl 2-methylbutyrate	Strong, green, fruity, apple odor and taste; also, some strawberry notes	Sweet, green apple, winey-fruity	20.0	0.46
Ethyl hexanoate	Strong, fruity, pineapple, banana with strawberry, pear and tropical notes	Weak fruity, sweet	15.1	0.15
Hexyl acetate	Sweet, fruity, pear-apple-like odor; green, banana, apple-pear-like taste	Heavy sweet, fruity	12.4	0.13
Methyl cinnamate	Fruity-balsamic odor; sweet fruity (cherry-strawberry) taste	Spicy, fruity, balsamic, sweet, green, floral	10.7	0.20
(Z)-3-Hexenol	Strong, fresh, green, grassy odor	Green, leaf-like	21.8	0.40
gamma-Decalactone	Coconut-peach-like odor; in dilution, peach taste	Peach, sweet, smoothing	18.9	0.16
Acetic acid	Pungent, sour, vinegar odor with sour, acid taste	Pungent, acrid; sweet at 200 ppm	20.0	0.33
Butanoic acid	Strong, cheese, butter-like, sour-rancid odor and taste	Smoothing, buttery, fruity	15.1	0.16
2-Methylbutanoic acid	Pungent, acrid, Roquefort cheese like; fruity in dilution	Smoothing, cream, butter, nutty	11.3	0.23
Hexanoic acid	Heavy, fatty, cheesy-sweaty odor and taste	Smoothing, waxy, cream, maple notes	10.1	0.09
Dimethyl sulfide	Pungent, cabbage, cooked vegetable odor; corn-like on dilution	Minor components in fruit and rose flavors	6.1	0.03

**Figure 2:**
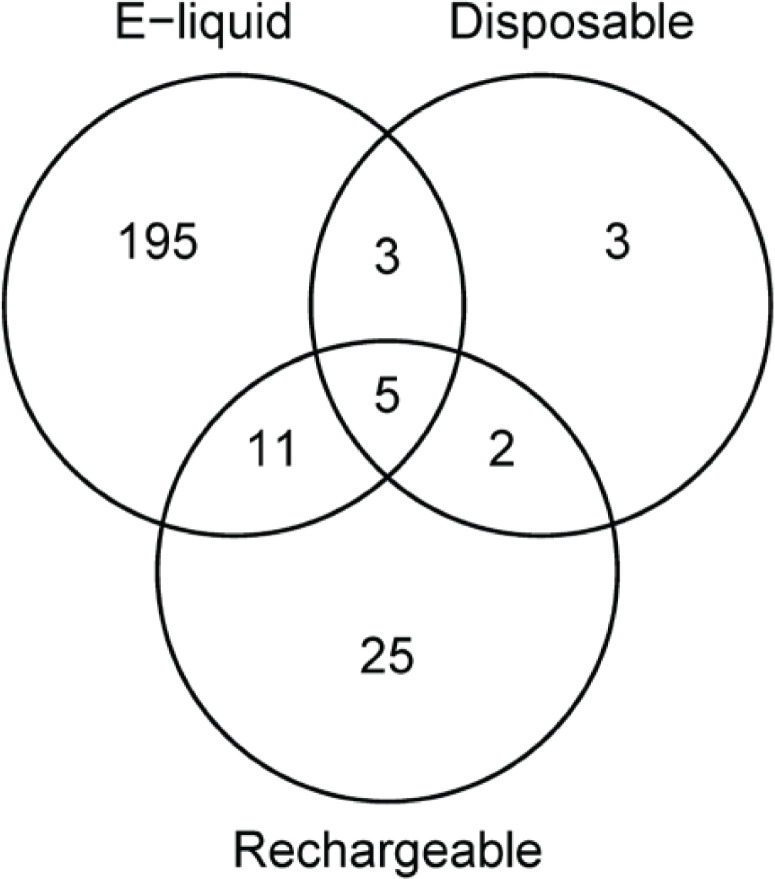
Venn diagram for ingredient combinations. The Venn diagram shows the degree of overlap between significantly co-occurring ingredient pairs in e-liquids, disposable and rechargeable e-cigarettes

The ingredient networks for disposables and rechargeables contained several pairs that were also found for the e-liquids, as can be seen from Supplementary file Figures 1 and 2, and is further illustrated as a Venn diagram in Figure 2. Many overlapping pairs involved one or two of the ingredients in clusters A or B. This applied, for example, to four ingredient pairs that were found in all three product types, namely: ethyl butyrate with ethyl hexanoate, isopentyl acetate and ethyl 2-methylbutyrate, and 2-methylbutanoic acid with ethyl 2-methylbutyrate. Other ingredient pairs that were found in multiple product types but did not involve ingredients from either cluster were linalool and beta-ionone, both of which showed a high use prevalence in berry-flavored e-liquids in a previous study from our team^12^, and cyclotene with 2,3,5-trimethyl pyrazine as well as 2-acetyl pyrazine, which were frequently used in tobacco- and nut-flavored e-liquids^12^. Also, the combination nicotine–benzoic acid was found in all three product types.

## DISCUSSION

The flavor of e-liquids and related products plays an important role in their attractiveness. Although we previously found that the flavor category (such as tobacco, menthol, citrus, etc.) of an e-liquid can essentially be predicted based on the ingredients that it contains^12^, the presence of multiple flavorings would allow for flavor interactions to occur. This would add complexity to our interpretation of how e-liquids are experienced by their users. To improve our understanding of e-liquid compositions, we determined if we could identify ingredient pairs that were frequently used together. Our results show that, indeed, such pairs can be found and that they form a network that tends to group ingredients with similar flavors or chemical structures together. Moreover, we identified two network clusters with denser connectivity, which will be discussed in more detail further below.

Our analysis started by determining the degree of overrepresentation of an ingredient pair compared to the prevalence of the two individual ingredients. Because there are many ingredients present in e-cigarette products and the number of potential pairs for *n* ingredients equals *n*(*n*-1)/2, the number of potential pairs becomes so large that it would be easy to accumulate false positive findings. To prevent this, we set a lower limit to the prevalence of use so that in the case of e-liquids, this led to 194 ingredients out of a total of 1779 notified ingredients being included for analysis. As this still allows for 194×193/2 = 18721 ingredient pairs, a stringent odds ratio for overrepresentation was set. A robustness analysis with randomized data found no significantly overrepresented ingredient pairs, and therefore, the contribution of false positive findings to our results should be negligible.

Among the product types examined, the e-liquid network (Figure 1) formed the largest network. Networks for disposables and rechargeables were smaller and less intricate. As the e-liquid ingredient network was the most informative and the number of e-liquids notified for the Dutch market is far larger than that for the other product types examined, further analyses were focused on this product type.

The network visualization layout algorithm only uses a list of ingredients and their connections as input, and arranges these connections in a two-dimensional graph using an optimization method that is described in more detail elsewhere^22,23^. Therefore, the algorithm is agnostic to the chemical or flavor properties of the ingredients, and the proximity of two ingredients in the network graph will depend partly on whether they are directly connected, as well as on whether they share (direct or indirect) connections to other ingredients. Indeed, applying the layout algorithm to the e-liquid ingredient network resulted in a graph in which ingredients with similar (flavor or chemical) properties are often positioned near each other, even if they do not share a direct connection (Figure 1). Overall, the e-liquid ingredient network shows sweet-creamy flavors in the top left area (e.g. vanillin, gamma-nonalactone, delta-decalactone), baked-roasted flavors in the lower left area (e.g. pyrazines, cyclotene, guaiacol), fruity flavors in the center and fruity-flowery flavors (e.g. geraniol, alpha- and beta-ionone) towards the right side of the main network. This illustrates how network analysis software can help discover patterns in a list of over 200 ingredient pairs.

To further ascertain underlying patterns, we applied cluster analysis to the network. This resulted in one cluster (cluster A) with ingredients that had, as expected, similar flavors that can be summarized as sweet-vanilla-creamy. There can be several reasons why these flavorings are used together. For example, besides vanillin, extracts of natural and artificial vanilla flavor often contain other substances such as ethyl vanillin, piperonal, and anisaldehyde^26^. Accordingly, these flavorings may help to get a more realistic vanilla flavor. Additionally, piperonal (also known as heliotropin), anisaldehyde, delta-decalactone, and gamma-nonalactone are often used in vanilla-flavored dairy products^27^ so their combined use in e-liquids may be to give users a taste they are already familiar with from a food source – and familiarity increases liking^28^. The composition of cluster B was somewhat counterintuitive as it contained, among others, fruity-smelling esters but also alkyl carboxylic acids and dimethyl sulfide, with the last two having unpleasant flavor descriptions. Cluster B contained four alkyl carboxylic acids, which have sour and/or cheesy flavors. For e-liquids, such flavors by themselves would likely be unattractive. However, according to the Leffingwell database^25^, these alkyl carboxylic acids also add a smoothing, creamy, or even sweet flavor to tobacco smoke. Butyric and hexanoic acid, along with gamma-decalactone, which is also found in cluster B, have been described as useful additions to foodstuffs to help achieve a rounded and fuller, creamier sensory profile^27^. It seems reasonable to assume that they contribute in a similar way to the flavor of e-liquids when inhaled as a mixture in a vapor or aerosol. Also, in a study that compared e-liquids between different flavor categories, butyric acid, and hexanoic acid, showed the highest prevalence in dessert-flavored e-liquids, followed by berry-flavored e-liquids; acetic acid showed less pronounced differences between flavor categories but the highest prevalence was found in berry-flavored e-liquids^12^. Hence, the use of alkyl carboxylic acids may be explained by their contribution to a creamy and/or fruity flavor. Dimethyl sulfide was another ingredient in cluster B that had an unpleasant flavor (cabbage, cooked vegetable). Nevertheless, its use in 6.1% of all e-liquids points to a crucial role in (at least some) e-liquid aromas. Comparing e-liquid ingredients between different flavor categories showed that dimethyl sulfide has the highest prevalence in berry-flavored e-liquids^12^. Also, studies have found that dimethyl sulfide, at low levels, enhanced the fruity notes of Syrah and Grenache Noir wines^29^ and in a model solution modulated the blackberry and enhanced the blackcurrant aroma of red wine fruity esters^30^. These berry-related associations would be in agreement with the fact that, in addition to six ingredients in cluster B, dimethyl sulfide also significantly co-occurs with beta-ionone, linalool, furaneol, raspberry ketone, and (Z)-3-hexenyl acetate (Figure 1), all of which have fruity flavors. Additionally, a study on dark chocolate samples found that the perception of both bitterness and astringency increases with higher concentrations of dimethyl sulfide. The authors of this study suggest that the unpleasant notes of dimethyl sulfide are masked by more pleasant notes, such as sweetness associated with other components^31^. Taken together, these findings indicate that dimethyl sulfide plays a role in refining sweet and/or fruity flavors. Overall, this suggests that the flavorings in cluster B act in concert, with some providing a fruity flavor (esters and gamma-decalactone), some adding a smoothing, creamy, sweet flavor (alkyl carboxylic acids and gamma-decalactone), and some additional refinement ((Z)-3-hexenol and dimethyl sulfide).

There are five ingredient pairs that significantly co-occurred in three product types. One of these pairs is nicotine with benzoic acid. This combination can be explained by the use of benzoic acid to protonate nicotine and make it less harsh^32,33^. We did not find significant overrepresentation for combinations of nicotine with other known nicotine protonating agents, such as lactic acid, mainly as a result of our stringent criteria regarding minimal prevalence and overrepresentation. However, for disposables, we also found significant overrepresentation for the combination of nicotine with 2-methylhexanoic acid. Although this ingredient will also protonate nicotine, it cannot be concluded from our data alone that it is used for this purpose or that its flavor (‘fatty-cheese-fruity, sour odor; creamy, fruity, nutty taste’^25^) may, for example, mask that of nicotine. The fact that nicotine 2-methylhexanoate, i.e. the nicotine salt resulting from these two ingredients, is currently not notified in EU-CEG suggests that this ingredient pair occurs for another reason or might even be undeliberate.

Our study was performed using data as they were notified of products before the flavor ban officially took effect. Because there was a one-year transition period during which e-liquids are still allowed on the market, it can be expected that the data and our results are not affected by the flavor ban. If we speculate on what results we would find if we repeat this study in the future, it can be expected that most ingredient pairs would not be found anymore because they involve flavorings that will no longer be allowed. However, it can be expected that manufacturers will compose new e-liquids consisting of new combinations of flavorings as a response to the Dutch flavor ban. Of the 16 flavorings that will be allowed under the flavoring ban in the Netherlands, only damascenone showed co-occurrence with other ingredients, namely with nicotine and furaneol (taste description: fruity, caramelized pineapple-strawberry; roasted), both in rechargeable e-cigarettes (Supplementary file Figure 2). This limited number of combinations and the fact that furaneol is a flavoring that will no longer be allowed suggests that interactions between allowed flavorings resulting in non-tobacco flavors will probably not be a significant concern in the near future. However, monitoring the composition and flavor of new products will remain necessary to enforce the flavor ban.

### Strengths and limitations

This study provides an example of how big data analyses can help to gain new insights from existing databases by identifying underlying patterns. For our analyses, we used data available in EU-CEG. These data are submitted to EU-CEG by manufacturers as part of their mandatory reporting to national authorities. It is not always possible or feasible to ensure the data are complete, current, and correct. Also, as our data set is based on the Dutch market at the time of analysis, there may be flavor (ing) interactions that were not found because the resulting flavor does not have sufficient appeal to Dutch e-cigarette consumers. An example of this would be the pairing of ethyl 2-methylbutyrate (fruity) and 1-(ethylsulfanyl)ethane-1-thiol (roasted onion), which would result in a durian aroma^34^. Furthermore, some practical choices made during the analysis, for example, the use of network visualization rather than multidimensional scaling, could have influenced the results obtained. Given these limitations, some caution is needed when interpreting the data. Finally, translating the mathematical results into meaningful results from, for example, a flavor science or legislation point of view, requires expert knowledge from these respective fields.

## CONCLUSIONS

Overall, our results show that flavor interactions play a role in the composition of e-liquids and that some ingredient combinations can create flavors that are different from those of the individual ingredients. For regulation regarding product attractiveness, such as a flavor ban, this implies that monitoring the composition and flavor of new products will remain essential, to ensure that new e-liquid products comply with the overall aims of e-cigarette legislation.

## Data Availability

The data supporting this research cannot be made available for privacy or other reasons. Some of the data are considered business confidential as they involve product compositions.

## References

[1] Scientific Committee on Health, Environmental and Emerging Risks (SCHEER). Opinion on electronic cigarettes. 2021. Accessed November 24, 2023. https://ec.europa.eu/health/sites/health/files/scientific_committees/scheer/docs/scheer_o_017.pdf 10.1186/s12954-021-00476-6 PMC7945356 33691708

[2] Glantz SA, Bareham DW. E-cigarettes: use, effects on smoking, risks, and policy implications. Annu Rev Public Health. 2018;39:215-235. doi:10.1146/annurev-publhealth-040617-013757 29323609 PMC6251310

[3] Patten T, De Biasi M. History repeats itself: role of characterizing flavors on nicotine use and abuse. Neuropharmacology. 2020;177:108162. doi:10.1016/j.neuropharm.2020.108162 32497589 PMC7814940

[4] Becker TD, Rice TR. Youth vaping: a review and update on global epidemiology, physical and behavioral health risks, and clinical considerations. Eur J Pediatr. 2022;181(2):453-462. doi:10.1007/s00431-021-04220-x 34396473 PMC8364775

[5] Boer M, van Dorsselaer S, de Looze M, et al. HBSC 2021: Gezondheid en welzijn van jongeren in Nederland. Utrecht University; 2022. Accessed November 24, 2023. https://www.trimbos.nl/wp-content/uploads/2022/09/AF2022-HBSC-2021-Gezondheid-en-welzijn-van-jongeren-in-Nederland.pdf

[6] Laverty AA, Vardavas CI, Filippidis FT. Design and marketing features influencing choice of e-cigarettes and tobacco in the EU. Eur J Public Health. 2016;26(5):838-841. doi:10.1093/eurpub/ckw109 27471217 PMC5054276

[7] Romijnders KA, Krüsemann EJ, Boesveldt S, Graaf K, Vries H, Talhout R. E-liquid flavor preferences and individual factors related to vaping: a survey among Dutch never-users, smokers, dual users, and exclusive vapers. Int J Environ Res Public Health. 2019;16(23):4661. doi:10.3390/ijerph16234661 31766776 PMC6926905

[8] Rijksoverheid. Smaakjes van e-sigaretten worden verboden. 2020. Accessed November 24, 2023. https://www.rijksoverheid.nl/actueel/nieuws/2020/06/23/smaakjes-van-e-sigaretten-worden-verboden

[9] Staatscourant. Regeling van de Staatssecretaris van Volksgezondheid, Welzijn en Sport van 22 november 2022, kenmerk 3456548-1038502-WJZ, houdende wijziging van de Tabaks- en rookwarenregeling ter regulering van smaken voor e-sigaretten. 2022. Accessed November 24, 2023. https://zoek.officielebekendmakingen.nl/stcrt-2022-32367.pdf

[10] Pennings JLA, Havermans A, Krusemann EJZ, et al. Reducing attractiveness of e-liquids: proposal for a restrictive list of tobacco-related flavourings. Tob Control. 2023. doi:10.1136/tc-2022-057764 PMC10958261 36669881

[11] Bartoshuk LM, Murphy C, Cleveland CT. Sweet taste of dilute NaCl: psychophysical evidence for a sweet stimulus. Physiol Behav. 1978;21(4):609-613. doi:10.1016/0031-9384(78)90138-5 740780

[12] Krüsemann EJZ, Havermans A, Pennings JLA, de Graaf K, Boesveldt S, Talhout R. Comprehensive overview of common e-liquid ingredients and how they can be used to predict an e-liquid's flavour category. Tob Control. 2021;30(2):185-191. doi:10.1136/tobaccocontrol-2019-055447 32041831 PMC7907577

[13] Moio L, Langlois D, Etievant PX, Addeo F. Powerful odorants in water buffalo and bovine mozzarella cheese by use of extract dilution sniffing analysis. Ital J Food Sci 1993;3:227–237.

[14] Burgard DR. Chemometrics: Chemical and Sensory Data. CRC Press.1990. doi:10.1201/9781351070607

[15] Grabenhorst F, Rolls ET, Margot C. A hedonically complex odor mixture produces an attentional capture effect in the brain. Neuroimage. 2011;55(2):832-843. doi:10.1016/j.neuroimage.2010.12.023 21168513

[16] Green BG, Lim J, Osterhoff F, Blacher K, Nachtigal D. Taste mixture interactions: suppression, additivity, and the predominance of sweetness. Physiol Behav. 2010;101(5):731-737. doi:10.1016/j.physbeh.2010.08.013 20800076 PMC2975745

[17] Carstens E, Carstens MI. Sensory effects of nicotine and tobacco. Nicotine Tob Res. 2022;24(3):306-315. doi:10.1093/ntr/ntab086 33955474 PMC8842437

[18] Leventhal AM, Tackett AP, Whitted L, Jordt SE, Jabba SV. Ice flavours and non-menthol synthetic cooling agents in e-cigarette products: a review. Tob Control. 2023;32(6):769-777. doi:10.1136/tobaccocontrol-2021-057073 35483721 PMC9613790

[19] European Commission. EU common entry gate (EU-CEG). Accessed November 24, 2023. https://ec.europa.eu/health/euceg/

[20] European Union. Directive 2014/40/EU of the European Parliament and of the Council of 3 April 2014 on the approximation of the laws, regulations and administrative provisions of the Member States concerning the manufacture, presentation and sale of tobacco and related products and repealing Directive 2001/37/EC Text with EEA relevance. 2014. Accessed November 24, 2023. http://data.europa.eu/eli/dir/2014/40/oj

[21] Szumilas M. Explaining odds ratios. J Can Acad Child Adolesc Psychiatry. 2010;19(3):227-229. 20842279 PMC2938757

[22] Shannon P, Markiel A, Ozier O, et al. Cytoscape: a software environment for integrated models of biomolecular interaction networks. Genome Res. 2003;13(11):2498-2504. doi:10.1101/gr.1239303 14597658 PMC403769

[23] Kamada T, Kawai S. An algorithm for drawing general undirected graphs. Inf Process Lett 1989;31(1):7-15. doi:10.1016/0020-0190(89)90102-6

[24] Bader GD, Hogue CW. An automated method for finding molecular complexes in large protein interaction networks. BMC Bioinformatics. 2003;4:2. doi:10.1186/1471-2105-4-2 12525261 10.1186/1471-2105-4-2 PMC149346

[25] Leffingwell & Associates. Flavor-Base, 9th Edition. Leffingwell & Associates, Inc. 2013.

[26] Sujalmi S, Suharso , Supriyanto R, Buchari. Determination of vanillin in vanilla (Vanilla planifolia Andrews) from Lampung Indonesia by high performance liquid chromatography. Indo J Chem 2005;5(1):7-10. doi:10.22146/ijc.21831

[27] Reiss I, Gatfield I-L, Krammer G, Clerc A, Kindel G, inventors. Use of divanillin as a flavouring agent. US patent application US 2006/0286237 A1. 2006.

[28] Pula K, Parks CD, Ross CF. Regulatory focus and food choice motives. Prevention orientation associated with mood, convenience, and familiarity. Appetite. 2014;78:15-22. doi:10.1016/j.appet.2014.02.015 24583413

[29] Segurel MA, Razungles AJ, Riou C, Salles M, Baumes RL. Contribution of dimethyl sulfide to the aroma of Syrah and Grenache Noir wines and estimation of its potential in grapes of these varieties. J Agric Food Chem. 2004;52(23):7084-7093. doi:10.1021/jf049160a 15537322

[30] Lytra G, Tempere S, Zhang S, et al. Olfactory impact of dimethyl sulfide on red wine fruity esters aroma expression in model solution. J Int Sci Vigne Vin 2014;48(1):75-85. doi:10.20870/oeno-one.2014.48.1.1660

[31] Guzman Penella S, Boulanger R, Maraval I, et al. Link between flavor perception and volatile compound composition of dark chocolates derived from Trinitario cocoa beans from Dominican Republic. Molecules 2023;28(9). doi:10.3390/molecules28093805 PMC10180179 37175215

[32] Leventhal AM, Madden DR, Peraza N, et al. Effect of exposure to e-cigarettes with salt vs free-base nicotine on the appeal and sensory experience of vaping: a randomized clinical trial. JAMA Netw Open. 2021;4(1):e2032757. doi:10.1001/jamanetworkopen.2020.32757 33433597 PMC7804919

[33] Duell AK, Pankow JF, Peyton DH. Nicotine in tobacco product aerosols: 'It's déjà vu all over again'. Tob Control. 2020;29(6):656-662. doi:10.1136/tobaccocontrol-2019-055275 31848312 PMC7591799

[34] Li JX, Schieberle P, Steinhaus M. Insights into the key compounds of Durian (Durio zibethinus L. 'Monthong') pulp odor by odorant quantitation and aroma simulation experiments. J Agric Food Chem. 2017;65(3):639-647. doi:10.1021/acs.jafc.6b05299 28024392

