# Supplementary material for: An ingredient co-occurrence network gives insight into e-liquid flavor complexity
Source: Tob Induc Dis. 2024 Jan 10;22:10.18332/tid/175955. doi: 10.18332/tid/175955 (PMC10777478; doi:10.18332/tid/175955)
Supplement: Supplementary file 1 [file TID-22-08-s1.pdf]

# Supplementary file

### Supplementary Example 1: co-occurrence analysis odds ratio

| | | Ethyl 2-methylbutyrate | |
|---|---|---|---|
| | | absent | present |
| Dimethyl sulfide | absent | 25967 | 5199 |
| | present | 566 | 1447 |

In this example, there are 33,179 e-liquids. Ethyl 2-methylbutyrate and dimethyl sulfide occur together in 1,447 of these, which is 4.4% of all liquids. The odds for finding ethyl 2-methylbutyrate in an e-liquid that *does not* contain dimethyl sulfide is 5,199/25,967 = 0.20. The odds for finding ethyl 2-methylbutyrate in an e-liquid that *does* contain dimethyl sulfide is 1,447/566 = 2.56. Hence the odds ratio = 2.56/0.20 = 12.8.

**Supplementary Figure 1. Ingredient network for disposable e-cigarettes.** Connections indicate significantly overrepresented ingredient combinations. Orange, cluster B; blue, other flavorings; pink, nicotine. Bold lines indicate combinations found for multiple product types.

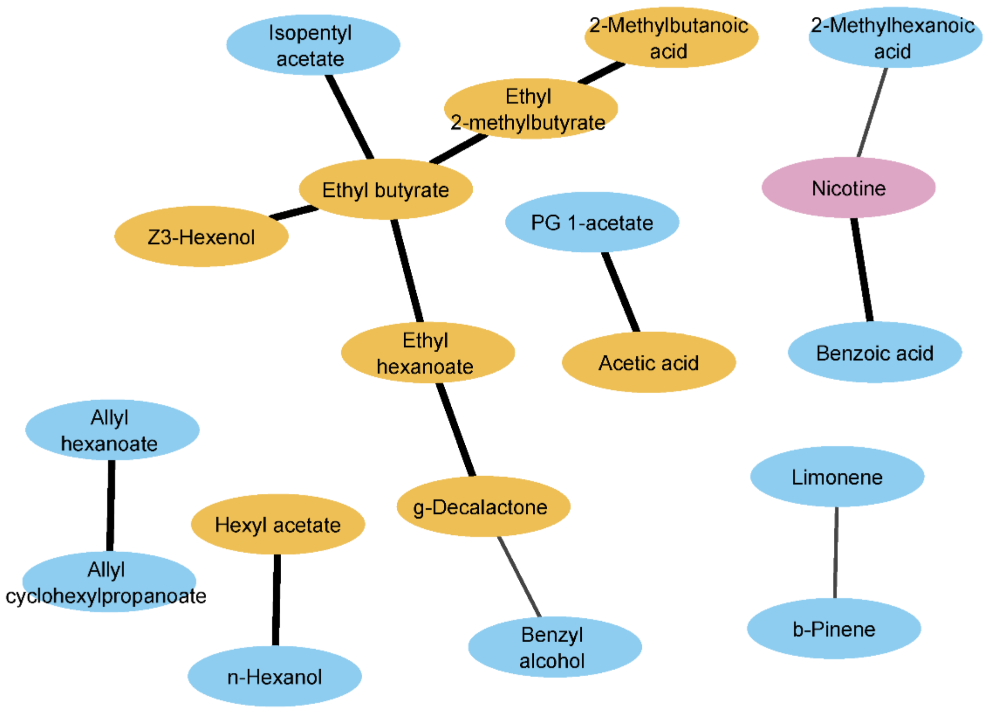

**Supplementary Figure 2. Ingredient network for rechargeable e-cigarettes.** Connections indicate significantly overrepresented ingredient combinations. Green, cluster A; orange, cluster B; blue, other flavorings; pink, nicotine or related . Bold lines indicate combinations found for multiple product types.

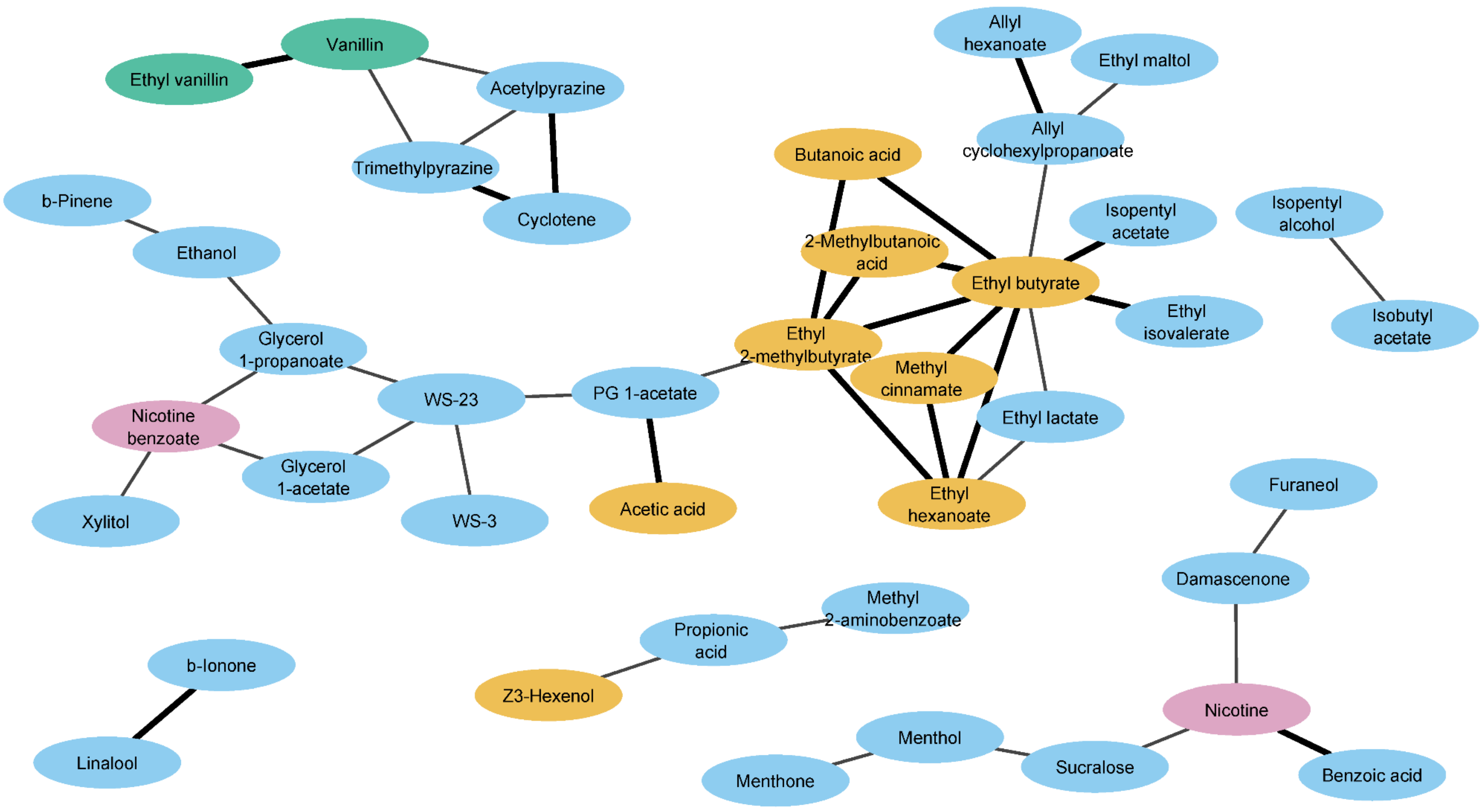

**Supplementary Figure 3. E-liquid ingredient network clusters A (left) and B (right).** Connections indicate significantly overrepresented ingredient combinations. Bold lines indicate combinations found for multiple product types.

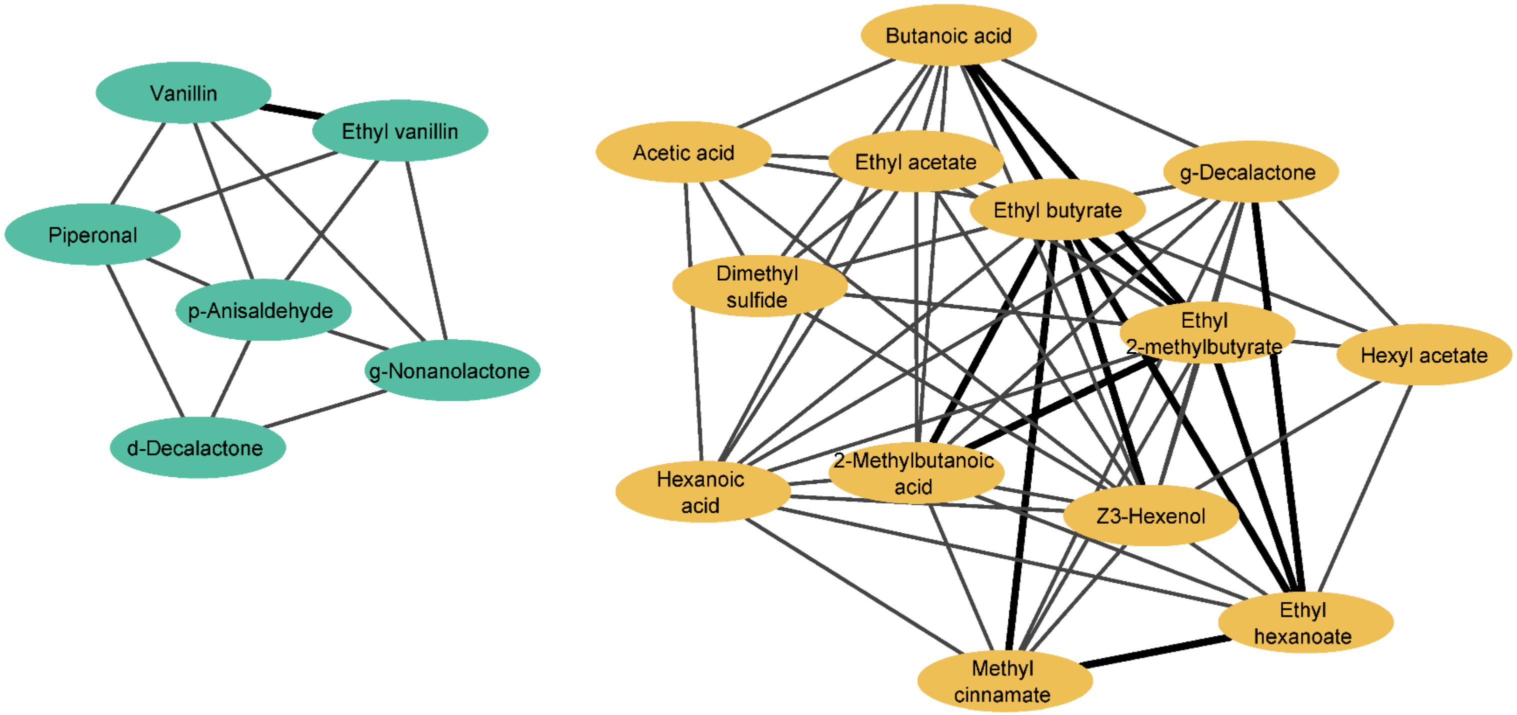


© 2024 Pennings J.L.A. et al.
